# 外周血宏基因组二代测序技术在血液病合并发热患者中的临床应用价值

**DOI:** 10.3760/cma.j.issn.0253-2727.2022.09.009

**Published:** 2022-09

**Authors:** 山凤 郝, 一浩 王, 丽娟 李, 化泉 王, 嘉 宋, 玉红 吴, 文 瞿, 国锦 王, 晓明 王, 鸿 刘, 莉民 邢, 晶 关, 宗鸿 邵, 蓉 付

**Affiliations:** 天津医科大学总医院血液内科，天津 300052 Department of Hematology, Tianjin Medical University General Hospital, Tianjin 300052, China

**Keywords:** 宏基因组二代测序, 血液病, 临床认可度, Metagenomic next generation sequencing, Hematological diseases, Clinical adjudication

## Abstract

**目的:**

探讨外周血宏基因组二代测序技术（mNGS）在血液病合并发热患者中的临床应用价值。

**方法:**

回顾性分析2020年3月至2021年6月在天津医科大学总医院血液科住院治疗的血液病合并发热并进行外周血mNGS检测的90例患者共98份标本的mNGS结果及临床资料，分析病原分布特征与血mNGS的检验效能。

**结果:**

外周血mNGS阳性率为68.37％（67/98），明显高于传统检查（37.76％，*P*<0.001）与血培养（9.18％，*P*<0.001）。单纯检出病毒、细菌、真菌阳性样本分别占38.81％、14.93％、2.99％；混合感染占43.28％，其中以病毒和细菌混合型最为多见（25.37％）；病毒阳性55例次（82.09％），细菌阳性30例次（44.78％），真菌阳性14例次（20.90％）。外周血mNGS与传统检查的临床认可率为64.63％（63/98）；以传统检查结果作为参照标准，外周血mNGS敏感性、特异性、阳性预测值（PPV）和阴性预测值（NPV）分别为75.68％、36.07％、41.79％和70.97％，整体一致率为51.02％。传统检查阴性的22例次肺部感染，有14例次外周血mNGS检出病原，其中10例次为临床认可。

**结论:**

该组血液病合并发热患者外周血mNGS病毒检出率高、混合感染比例高；外周血mNGS阳性率明显高于血培养以及传统实验室检查；外周血mNGS在血液病合并发热患者病原检出方面具有较高的临床认可率、敏感性及阴性预测值。

## Abstract (en)

**Objective:**

To investigate the clinical application value of peripheral blood metagenomic next-generation sequencing（mNGS）test for patients with hematological diseases accompanied by fever.

**Methods:**

The blood mNGS results and clinical data of inpatients with hematological diseases accompanied by fever treated in the Hematology Department of Tianjin Medical University General Hospital in March 2020 to June 2021were retrospectively analyzed. A total of 90 patients with 98 cases of specimens were included. The pathogen distribution characteristics and mNGS test performance were analyzed.

**Results:**

The positive rate of peripheral blood mNGS was significantly higher than that of traditional examination（68.37％ *vs* 37.76％, *P*<0.001）and blood culture（68.37％ *vs* 9.18％, *P*<0.001）. Viral, bacterial, and fungal infections accounted for 38.81％, 14.93％, and 2.99％ in patients with single-pathogen infections, respectively. Polymicrobial infections accounted for 43.28％, in which viral and bacterial coinfections were the most common type（25.37％）. There were 55 virus-positive cases（82.09％）, 30 bacteria-positive cases（44.78％）, and 14 fungus-positive cases（20.90％）. The clinical approval rate of peripheral blood mNGS was 64.63％（63/98）. The sensitivity, specificity, positive predictive value, and negative predictive value（NPV）of peripheral blood mNGS were 75.68％, 36.07％, 41.79％, and 70.97％, respectively, and the overall consistency rate with traditional examination was 51.02％. Of the 22 pulmonary infection cases with no detectable pathogens by conventional tests, the pathogens were identified by peripheral blood mNGS in 14 cases, 10 of which were clinically approved.

**Conclusion:**

The positive rate of peripheral blood mNGS was significantly higher than that of blood culture and traditional laboratory examination. Peripheral blood mNGS had a high clinical recognition rate, sensitivity, and NPV in the detection of pathogens in patients with hematological diseases accompanied by fever.

感染是导致恶性血液病患者死亡的重要原因之一，以肺部感染、血流感染较为常见[1]。由于单纯血培养检测阳性率低且及时性差，且血液病患者支气管镜等有创操作的应用受限，因此需要一种快速、无创、广谱而又精准的方法确定感染原。宏基因组二代测序技术（mNGS）检测具有无偏倚性、广覆盖、快速等优点，能覆盖更广范围的病原体[2]–[3]。2019年，首个商业化血浆mNGS检测（Karius检测）被证实可大幅提高脓毒症患者的病原体检出率[4]。随后多个研究也证实了血浆mNGS在真实世界的应用价值[5]–[7]。目前外周血mNGS在成人血液病患者中的临床应用价值探讨尚少[8]。本研究中，我们回顾性分析2020年3月至2021年6月98例次外周血mNGS结果，旨在了解外周血mNGS的病原谱特征、临床应用价值，观察外周血mNGS阳性与阴性患者的临床指标差异，力图寻找血液病患者进行外周血mNGS检测的适宜人群和时机。

## 病例与方法

1. 病例资料：以2020年3月至2021年6月天津医科大学总医院血液科住院治疗的血液病合并发热并送检外周血mNGS（DNA）检测的90例患者共计98份标本（同一患者两次外周血mNGS检测间隔大于15 d）为研究对象。患者标本采集时均有发热，之前均进行了传统实验室检查未获得明确的病原学证据或者结果未回且经验性抗感染治疗无效。

2. mNGS检测：所有样本均由北京金匙基因科技有限公司进行DNA-mNGS检测及生物信息学分析。测序长度SE50，测序数据量不低于30M Reads。通过bowtie2和人的参考基因组GRCh38进行比较去除人的参考基因组序列，采用SNAP与微生物基因组数据库进行比对，从而对微生物进行鉴定。

3. 传统实验室检查：包括血培养及其他无菌体液培养、皮肤软组织分泌物培养、痰培养、尿培养、病毒核酸检测、（1,3）-β-D-葡聚糖试验（G试验）、曲霉半乳甘露抗原试验（GM试验）、痰抗酸染色、血结核杆菌斑点试验（T-SPOT）、痰X-PERT。

4. 临床认可与否的评判标准：参考文献[9]–[10]中对mNGS临床认可度评价方法制定了本中心临床认可与否的评判标准，由至少3名专家，包括血液内科、感染科、感染部位相关专业专家（如肺部感染患者由呼吸内科及影像科专家共同对mNGS结果进行临床判定），各专业进行临床评判的人员均固定，最大程度避免同专业不同人员间主观判断的差异对结果的影响。临床判定分为以下10种情况：①临床认可：mNGS阳性，与传统实验室检查阳性结果一致，证实了感染病原。②临床认可：mNGS阳性，与传统实验室检查阳性结果部分一致，即除了检出一致的病原外，mNGS检出了另外其他的疑似病原，且针对性的前期经验用药或后调整用药有效（mNGS覆盖广）。③临床认可：mNGS阳性，传统实验室检查阴性，参考mNGS阳性结果针对性用药有效（mNGS阳性率更高）。④临床认可：mNGS阴性，传统实验室检查阴性，符合临床预期（排除感染作用）。⑤临床认可：mNGS阳性，传统实验室检查阳性，阳性结果不一致，但参考mNGS阳性结果针对性用药有效。⑥意义不明确：mNGS阳性/阴性，传统实验室检查阴性，临床意义不明确，需结合患者转归再分为临床认可或不认可。⑦临床不认可：mNGS阳性，与传统实验室检查阳性结果不一致，不符合临床预期。⑧临床不认可：mNGS阴性，传统实验室检查阳性，两者结果不一致。⑨临床不认可：mNGS阳性，传统实验室检查阴性，临床不认可mNGS阳性结果。⑩临床不认可：mNGS阴性，传统实验室检查阴性，与临床诊断为感染不符。

5. 统计学处理：采用SPSS13.0进行统计分析。连续变量的两组间比较采用非参数秩和检验，分类变量的组间比较采用Fisher精确概率法。*P*<0.05为差异有统计学意义。

## 结果

1. 患者基本特征：90例患者中，女37例、男53例，中位年龄57.5（9～78）岁；55例（56.12％）患者感染部位为肺部。病种分布情况见表1，恶性血液病占73.33％。

**表1:** 90例血液病合并发热并送检外周血mNGS患者病种分布情况

疾病类型	例数	构成比（％）
恶性血液病	66	73.33
急性白血病	26	28.89
淋巴瘤	16	17.78
MDS或MDS/MPN	13	14.44
MM	7	7.78
CLL	2	2.22
CML	1	1.11
T-LGLL	1	1.11
非恶性血液病	24	26.67
SAA	5	5.56
感染相关血细胞减少	5	5.56
HLH	4	4.44
菊池病	2	2.22
特发性嗜酸性粒细胞增多症	2	2.22
MGUS	2	2.22
范可尼贫血	1	1.11
急性造血功能停滞	1	1.11
淋巴结炎	1	1.11
ITP	1	1.11

注：mNGS：宏基因组二代测序；MDS：骨髓增生异常综合征；MPN：骨髓增殖性肿瘤；MM：多发性骨髓瘤；CLL：慢性淋巴细胞白血病；CML：慢性髓性白血病；T-LGLL：T细胞大颗粒淋巴细胞白血病；SAA：重型再生障碍性贫血；HLH：噬血细胞性淋巴组织细胞增多症；MGUS：意义未明单克隆免疫球蛋白血症；ITP：免疫性血小板减少症

2. 外周血mNGS病原检测结果：98例次标本中共67例次外周血mNGS检出病原，单纯病毒、细菌、真菌阳性样本分别占38.81％、14.93％、2.99％；混合感染占43.28％，其中以病毒和细菌混合型感染最为多见（25.37％）；病毒阳性55例次（82.09％），细菌阳性30例次（44.78％），真菌阳性14例次（20.90％），巨细胞病毒、细环病毒和EB病毒为检出率较高；细菌以肺炎克雷伯菌检出率最高，其次为嗜麦芽窄食单胞菌、嗜肺军团菌、屎肠球菌；真菌种类无明显聚集性。

3. 外周血mNGS的检验效能：本组外周血mNGS阳性率为68.37％（67/98），传统检查阳性率为37.76％（37/98），血培养阳性率为9.18％（9/98），mNGS阳性率明显高于传统检查及血培养（*P*值均<0.001）。9份血培养阳性标本中，4例标本mNGS与血培养一致，临床认可。4例与血培养不一致，其中1例mNGS结果为黄曲霉，与临床表现一致因此判定为临床认可；其余3例临床不认可；1例mNGS阴性，临床不认可（表2）。

**表2:** 9例次血培养阳性标本的外周血mNGS以及临床认可度结果

序号	疾病类型	ANC（×10^9^/L）	血培养	外周血mNGS	结果是否一致	临床是否认可
1	AML	0.07	嗜麦芽窄食单胞菌	嗜麦芽窄食单胞菌+奇异变形杆菌	是	认可
2	ALL	1.14	屎肠球菌	屎肠球菌	是	认可
3	AML	0.01	嗜麦芽窄食单胞菌	嗜麦芽窄食单胞菌+阴沟肠杆菌	是	认可
4	AML	0.02	肺炎克雷伯菌	蜡样芽孢杆菌+细环病毒+HSV1+CMV	否	不认可
5	SAA	0.02	嗜麦芽窄食单胞菌	HSV1+EBV+CMV+人多瘤病毒	否	不认可
6	MDS	0.21	肺炎克雷伯菌	阴性	否	不认可
7	AML	0.04	盲肠肠球菌	黄曲霉菌	否	认可
8	CLL	1.38	沙门菌群	细环病毒+人多瘤病毒+HSV1	否	不认可
9	AML	0.05	铜绿假单胞菌+金黄色葡萄球菌	铜绿假单胞菌+金黄色葡萄球菌+细环病毒+HSV1+CMV+HHV-7	是	认可

注：mNGS：宏基因组二代测序；AML：急性髓系白血病；ALL：急性淋巴细胞白血病；SAA：重型再生障碍性贫血；MDS：骨髓增生异常综合征；CLL：慢性淋巴细胞白血病；HSV：单纯疱疹病毒；EBV：EB病毒；CMV：巨细胞病毒；HHV：人类疱疹病毒

外周血mNGS与传统检查总体临床认可率为64.63％（63/98），其中mNGS阳性标本的临床认可率为85.07％（57/67），mNGS阴性标本的临床认可率为19.35％（6/31）。本研究显示以传统检查结果作为参照标准，外周血mNGS敏感性、特异性、阳性预测值（PPV）和阴性预测值（NPV）分别为75.68％、36.07％、41.79％和70.97％，整体一致率为51.02％。

进一步分析肺部感染的样本，传统实验室检查阳性率为60％（33/55），外周血mNGS阳性率为72.73％（40/55），差异无统计学意义（*P*＝0.158）。在22例次传统实验结果无法提供任何病原学依据的样本中，外周血mNGS显示14例次阳性，其中10例次为临床认可，包含3例次耶氏肺孢子菌、2例次巨细胞病毒、1例次结核分枝杆菌合并EB病毒、1例次肺炎克雷伯杆菌合并分枝横梗霉及病毒、1例次屎肠球菌合并乳明串珠菌、1例次嗜肺军团菌合并病毒、1例次马赛血液杆菌。

4. 差异分析：PCT升高组患者较PCT正常组患者细环病毒检出率更高［18.8％（3/16）对0（0/32），*P*＝0.032］；粒细胞缺乏（粒缺）组较非粒缺组患者嗜麦芽窄食单胞菌检出率更高［23.5％（4/17）对0（0/51），*P*＝0.003］；但是粒缺组CMV检出率高于非粒缺组［52.9％（9/17）对23.5％（12/51），*P*＝0.012］；CRP正常组患者较CRP升高组人类疱疹病毒1型检出率高［60.0％（3/5）对9.1％（3/33），*P*＝0.021］。其他种类病原检出率在不同临床分组中差异均无统计学意义。

## 讨论

感染是血液病患者最常见的并发症及引起死亡的主要原因之一，及时发现致病微生物至关重要[1]。传统的血培养阳性率低，培养周期长，尽管粒缺伴发热患者血培养的阳性率较非粒缺伴发热患者高，但也仅有10％～25％[11]–[12]，且血培养无法检出病毒，真菌检出率低[13]。本研究外周血mNGS检出真菌阳性14例次，阳性率为20.9％，明显高于血培养。

杨理等[14]回顾性分析了41例血液病患者外周血mNGS数据，阳性率为58.54％，病毒与细菌检出率显著高于真菌检出率，混合感染以病毒和细菌混合感染最为多见。本研究结果与其基本一致。有研究表明免疫抑制的患者容易出现多重感染[15]，本研究73.3％的患者为恶性血液病，其余非恶性血液病患者也大部分伴粒缺，且大多长期使用免疫抑制剂，外周血mNGS显示近一半的样本存在混合感染，提示外周血mNGS在混合病原微生物的检出中具有一定优势。

另外一项研究[16]纳入的同样是免疫抑制的血液病患者，但是病毒检出率低于上述研究及本研究，可能原因是该研究的标本只有一部分是血液，其余为感染灶处的标本。我国一项对78例ICU患者外周血mNGS结果显示单纯病毒感染比例也高于细菌、真菌[17]；但是Jing等[18]对北京大学人民医院进行外周血mNGS检测的209份标本进行了分析，病毒检出率低于细菌检出率。血液病患者外周血mNGS病毒检出率高是血液病患者群体的特有现象还是血液标本较其他标本有特殊之处还需要与非血液病患者外周血标本进行比较来证实。

本研究血培养阳性9例标本仅有4例外周血mNGS结果与其吻合，同样也有研究显示血培养阳性而外周血mNGS阴性或病原结果与血培养不一致[17]–[18]，这也印证了指南所建议的尽管外周血mNGS阳性率高于传统血培养，但是血培养仍是疑似血流感染患者必做的检查，血培养与外周血mNGS存在优势互补，但仍无法互相替代[20]。

考虑到作为血流感染病原诊断金标准的血培养阳性率低，在判断外周血mNGS检验效能时又缺乏特异性指标，多项研究也采用了传统微生物检测方法（包括痰培养、感染部位标本培养、G试验、GM试验、T-SPOT、CMV-PCR、EBV-PCR等传统方法）与mNGS进行比较检测其临床效能[7],[18],[21]–[22]。此外多项研究采用临床认可度评价方法对mNGS效能进行评价[9]–[10]。虽然结果评判存在一定的主观因素，但是在缺乏金标准的情况下也不失为一种有效的参照指标。

Benamu等[10]以临床认可度为评判标准对粒缺伴发热患者外周血mNGS结果进行评估，其敏感度、特异度、PPV和NPV分别为92％、70％、93％和63％。国内一项研究显示，以传统微生物检查为参照标准，外周血mNGS的敏感度、特异度、PPV和NPV分别为86.0％、75.6％、71.2％和88.6％；以临床认可度为参照标准，上述指标分别为87.1％、80.2％、77.9％和88.6％[18]。本研究的特异度及PPV远低于上述两项研究，可能原因是mNGS阳性标本中很大一部分传统实验室检查阴性，进一步分析原因与本中心外周血mNGS病毒检出率高有关系，而传统实验室检查对于病毒检测存在一定局限性，因此外周血mNGS检出病毒阳性的标本缺乏有效的传统实验室检查为参照。所以若以传统实验室检查结果作为参照标准，传统检查是否全面，标本采集是否合格，所在中心检验的准确性等影响传统实验室检查的因素会直接影响到mNGS的检验效能；同理，如果以临床认可度作为参照，评判者的主观因素也会影响到mNGS的检验效能。本研究未使用临床认可有感染或无感染作为参照标准评价外周血mNGS检验效能，因为本研究中并未出现外周血mNGS阴性而临床研究者对此阴性结果判定为临床认可存在感染的情况。此外对mNGS阳性样本判定为临床认可无感染的情况本研究中也未遇到，此种情况本研究均判定为临床不认可，提示临床认可度判定标准很可能在不同中心存在差异。mNGS的检验效能评判难度较大，需要进一步细化参照标准才能保证各研究间的结果具有可比性。

血液科患者感染常发生于肺部[1]，本研究50％以上的病例存在肺部感染，按照指南推荐肺感染患者mNGS样本的最佳来源应是感染的组织或肺泡灌洗液[19],[22]，但很多血液病患者感染严重或不能耐受纤维支气管镜等有创操作，因此指南上建议可考虑采集患者的血液标本送检。本研究单独研究了肺部感染的样本，尽管通过痰培养、咽部拭子等传统方法检出的阳性率已达到60％，但是仍然有22例次样本传统方法无法提供任何病原学依据，而这些样本mNGS显示14例次阳性，其中10例次为临床认可，可见肺部感染患者传统微生物检验阴性的情况下，外周血mNGS检测的确是一个很好的补充。

本研究展示了单中心血液病合并发热患者外周mNGS病原分布情况及病毒检出率高、混合感染比例高的特点；mNGS阳性率明显高于血培养以及传统实验室检查，但仍无法取代血培养；本研究显示了外周血mNGS在血液病合并发热患者病原检出方面具有较高的临床认可率、敏感度及NPV，有较高的临床应用价值；对于传统实验室检查阴性的肺部感染患者，外周mNGS是有效的补充方法。mNGS凭借其诸多优点正快速地完成实验室到临床的转化，但适用人群和应用时机尚需进一步探索。
